# Depressive Symptoms among Middle-Aged Women—Understanding the Cause

**DOI:** 10.3390/brainsci11010026

**Published:** 2020-12-28

**Authors:** Anna M. Cybulska, Małgorzata Szkup, Daria Schneider-Matyka, Karolina Skonieczna-Żydecka, Mariusz Kaczmarczyk, Anna Jurczak, Sylwia Wieder-Huszla, Beata Karakiewicz, Elżbieta Grochans

**Affiliations:** 1 Department of Nursing, Faculty of Health Sciences, Pomeranian Medical University in Szczecin, 48 Żołnierska St., 71-210 Szczecin, Poland; anna.cybulska@pum.edu.pl (A.M.C.); malgorzata.szkup@pum.edu.pl (M.S.); daria.schneider-matyka@pum.edu.pl (D.S.-M.); grochans@pum.edu.pl (E.G.); 2 Department of Human Nutrition and Metabolomics, Pomeranian Medical University in Szczecin, 24 Broniewskiego St., 71-460 Szczecin, Poland; 3 Department of Clinical and Molecular Biochemistry Pomeranian Medical University in Szczecin, al. Powstancow Wlkp. 72, 70-111 Szczecin, Poland; mariush@pum.edu.pl; 4 Department of Specialized Nursing, Faculty of Health Sciences, Pomeranian Medical University in Szczecin, 48 Żołnierska St., 71-210 Szczecin, Poland; jurczaka@op.pl (A.J.); sylwia.huszla@pum.edu.pl (S.W.-H.); 5 Subdepartment of Social Medicine and Public Health, Department of Social Medicine, Faculty of Health. Sciences, Pomeranian Medical University in Szczecin, 48 Żołnierska St., 71-210 Szczecin, Poland; beata.karakiewicz@pum.edu.pl

**Keywords:** depressive symptoms, menopause, *MAO-A* gene, *5-HHT* gene

## Abstract

Menopause is an important event in a woman’s life associated with hormonal changes that play a substantial role in the functioning of her body. A decline in the level of estrogens contributes to depressive symptoms and mood disorders during this period. The severity of depressive symptoms experienced by middle-aged women depends on many factors, including sociodemographic data (e.g., menopause, employment status, and marital status) and genetic variables (*MAO-A* and *5-HTT* gene polymorphisms). In order to assess their influence on the development of depression in females, we analyzed 1453 healthy Polish women in different stages of menopause. Based on the results, we found that the l/l + l/s inheritance model for the *5-HTT* gene polymorphism was more common in women without and with moderate depressive symptoms according to the Beck Depression Inventory (BDI), while the l/s model was more often observed in women with mild depression. Moreover, the overdominant 3/3 + 4/4 genotype of the *MAO-A* gene polymorphism was more often found in respondents without depressive symptoms, while women with depressive symptoms had more often the overdominant 3/4 genotype.

## 1. Introduction

### 1.1. Menopause and Depression

According to the World Health Organization (WHO), natural menopause is the “permanent cessation of menstruation resulting from the loss of ovarian follicular activity” [1]. It has been divided into three stages, namely, premenopause―defined as the entire period of reproductive life before menopause; perimenopause―the immediate time before menopause with the symptoms of the approaching menopause (when the endocrine, biological, and clinical features of the approaching menopause begin) and the first year after menopause; and postmenopause―the time after the last menstruation (natural or artificial) [2,3].

Menopause is a natural part of life that impacts every woman as aging ensues [4]. It is an important event in a woman’s life, associated with the occurrence of symptoms such as hot flashes, night sweats, palpitations, mood swings, insomnia, anxiety, depression, concentration disorders, nervousness, headaches, mood lability, dysphoria, states of tension, and tearfulness [5]. Hormonal changes in the female body significantly affect women’s daily functioning. Estrogen, which is a mood modulator, plays a major role in depressive disorders in women, and its deficiency has a negative impact on their well-being [6].

Many epidemiological and clinical data confirm the view that from the age of ten, women suffer from depression more often than men [7,8,9]. This difference is most evident before the age of 45, when women seem to be the most vulnerable to depressive episodes [10]. Epidemiological studies indicate that approximately one out of five women will experience an episode of major depressive disorder (MDD) at some point in her life [11]. Depressive symptoms may develop or become more severe during the time of dynamic hormonal changes, such as the premenstrual, perinatal, or perimenopausal periods [12]. Studies show that the incidence of first or recurrent episodes of clinical depression in middle-aged women is 20–30% [13,14,15]. Some women are more sensitive to changes in sex hormone levels and, consequently, more susceptible to related negative mood symptoms and relapses of MDD, the risk of which increases in the perimenopausal period [16,17]. While some researchers suggest a 2–5-fold higher risk of depression in the perimenopausal period [16], others assert that the risk of depression and anxiety for women is high in both perimenopause and postmenopause [17,18]. There is much controversy over the contribution of menopause to the development of depression and anxiety. It is worth noting, however, that although the increased risk of clinical and subclinical depression is observed in the period of low estrogen status, its occurrence should not be directly attributed to menopause, but also neurotransmitters that play an important part in its development [19]. Depressive disorders are more likely to occur in women who develop vasomotor symptoms―among them hot flashes, night sweats, vaginal dryness, and dyspareunia―which negatively affect sleep [15]; conversely, depressive problems can worsen climacteric symptoms [20]. One should also not ignore other contributors, such as socio-economic data (marital status, education level, ethnic origin [21,22]), psychosocial, and psychological factors (lifestyle, body image, culture, and childhood stressors, i.e., abuse/neglect in youth, family problems, low socio-economic status, poverty, or dangerous living environment [18,21,23,24,25]), personality traits (pessimism, instrumentality [26,27,28,29]), and genetic determinants (MDD is inherited in approximately 35–40% of cases [30,31]) should also not be ignored [18,32,33,34].

### 1.2. Genetic Determinants of Depressive Disorders

Genetic factors may increase the risk of depressive symptoms [35]. Genes that may be involved in the development of psychiatric problems are *SLC6A4* encoding the serotonin transporter (5-HTT) and *MAOA* encoding the monoamine oxidase A (*MAO-A*) [36,37].

Currently, many studies in this field concern the functioning of the serotonergic system. 5-HTT is responsible for transport of serotonin from the synaptic cleft into presynaptic neurons. Changes in the transcriptional activity of the *5-HTT* gene are caused by the 44-bp insertion/deletion polymorphism in its promoter region (5-HTTLPR). The long (l) allele compared to the short (s) allele results in a much greater serotonin reuptake. This polymorphism has been widely studied because of the pleiotropic effect it can potentially have on mental functioning, personality traits and mental disorders in humans [38]. It has been proved that the “s” allele of the *5-HTT* gene (*SLC6A4*) is associated with lower *5-HTT* expression and function, thus causing transmission disturbances, which may result in higher impulsivity [39]. Moreover, carriers of the *SLC6A4* “s” allele are more likely to negatively react to stressful life events, which may cause serious depressive symptoms and even contribute to suicide attempts [40]. Some studies show that the decreased level of *5-HT* plays an important role in the development of depression [41,42]. On the other hand, excessive *5-HTT* activity may result in abnormally low extracellular serotonin levels, which in turn contributes to mood disorders and other mental problems, including depression [43].

Other studies suggest a positive relationship between the “s” allele and the *5-HTT* gene variant [44], and many studies emphasize that despite significant evidence for the role of serotonin in major depression, the findings regarding a possible association with the 5-HTTLPR allele are inconclusive, and there is no so far clear evidence for this relationship [45,46]. Enzymes involved in the regulation of 5-HT levels, such as monoamine oxidases (MAOs), may also play a significant role in the development of mental disorders. MAOs form a group of enzymes that catalyze the oxidative deamination of some neurotransmitters and dietary amines. Their expression largely depends on protein-coding genes, located on the X-chromosome [47]. *MAOA* has a polymorphism located 1.2 kb upstream of its coding sequence. It consists of a 30-bp repeated sequence present in 2 [48], 3, 3.5, 4, or 5 copies [49], the most common of which are the 3 and 4 alleles repeats [50]. The copy number affects the expression of the enzyme—the 3.5 and 4-repeat alleles have been shown to be expressed more efficiently than the others [48,49].

Due to its influence on the regulation of serotonin, dopamine, and noradrenaline levels, the *MAO-A* gene is considered to be an important factor contributing to individual differences in psychological characteristics and the severity of mental disorders [51,52,53,54].

The occurrence of depressive symptoms in menopausal women is associated with many factors, of which the most frequently documented are sociodemographic variables (age, professional activity) and genetic factors (*MAO-A* and *5-HTT* genes). Hence our decision to analyze the impact of selected genetic variables (*MAO-A* and *5-HTT* genes) and sociodemographic (age, place of residence, marital status, and employment status) on the risk of depression among Polish women in the pre-, peri-, and postmenopausal periods.

## 2. Materials and Methods

The study included 1500 healthy pre-, peri-, and postmenopausal women living in the West Pomeranian Voivodeship.

The criteria for inclusion in the study were:

- normal pap smear result;
- normal mammography result;
- normal blood pressure;
- no alcohol abuse history, no smoking;
- no history of selected endocrine disorders (thyroid diseases and diabetes);
- no history of cancerous diseases;
- no history of psychiatric treatment.

Patients were recruited through flyers placed in primary healthcare centers and specialist clinics in Szczecin. The study included 1453 women who met all inclusion criteria. The respondents were divided into three groups according to WHO division of the menopausal period. The following division was adopted [1]: premenopause (*n* = 247), perimeopause (*n* = 708), postmenopause (*n* = 498). The study was approved by the Bioethics Committee of the Pomeranian Medical University, Szczecin [KB-0080/187/09, KB-0012/104/11, KB-0012/12/12].

### 2.1. Procedure

#### 2.1.1. Preliminary Research

The research was carried out in several stages. After obtaining the written consent of the patient, we assessed her mental state using the Primary Care Evaluation of Mental Disorders Patient Health Questionnaire (PRIME-MD PHQ-9) for diagnosing mental diseases. The respondents who did not meet the inclusion criterion at this stage of the study were excluded.

#### 2.1.2. Survey

The next stage of the research was based on a survey performed using a standardized research tool, the Beck Depression Inventory (BDI), and the author’s questionnaire, which included a number of questions regarding demographic data (age, marital status, education, employment status, place of residence).

The BDI is a 21-question research instrument for measuring the severity of depression. Each question has a set of four possible responses, ranging in intensity (from 0 meaning the least severe to 3 meaning the most severe symptom). The sum of the points reflects the severity of depressive symptoms. The following score ranges were adopted in the study: no depression (0–11 points), mild depression (12–19 points), moderate depression (20–25 points), and severe depression (26 points and more) [55]. We also applied a more general division into patients without depression (less than 12 points) and with depression (12 points and more).

#### 2.1.3. Sampling and DNA Isolation from Whole Blood by Miller’s Salting-Out Method

In the next stage of the study, 10 mL blood samples were collected using the Vacutainer method. The patients were informed how they should be prepared for the study (they should be after a 12-h fast). Blood samples were collected at the blood collection point between 7.00 and 10.00 a.m. The biological material was stored and transported in accordance with the procedures of the quality management system of the Genetic Laboratory of the Department of Psychiatry, the Pomeranian Medical University, Szczecin (in accordance with the EN 15189 standard). The research was carried out in the Genetic Laboratory of the Department of Psychiatry, the Pomeranian Medical University, Szczecin.

DNA was isolated from whole blood by the Miller salting out method [56]. After obtaining DNA, the PCR method was used to identify DNA polymorphisms, thanks to which data on the polymorphisms of the MAOA and the 5-HTT genes were obtained. The following primer sequences were obtained for *MAO-A*: F: 5′ CCC AGG CTG CTC CAG AAA 3′ and MAO-A R: 5′ GGA CCT GGG TTG TGC 3′. The sizes of the amplified fragments were as follows: 239, 209, 226, and 269 bp. In the case of the 5-HTT gene polymorphism, the fragment including 44 bp ins/del in the regulatory sequence was amplified. The primer sequences were as follows: HTT F: 5′ GGC GTT GCC GCT CTG AAT GC 3′ and HTT R: GAG GGA CTG AGC TGG ACA ACC AC 3′. The sizes of the amplified fragments were as follows: 484 and 528 bp.

#### 2.1.4. Statistical Analysis

The results obtained were statistically analyzed using the MedCalc ver. 19.2 (Osten, Belgium). The normality of the variable distribution was tested using the Shapiro–Wilk test. Consequently, as the distribution of continuous variables differed from the normal distribution these were expressed as a median and interquartile range. For qualitative variables, a number was given, also expressed as a percentage. Differences in sociodemographic and anthropometric variables were analyzed using the Mann–Whitney *U* test or the Kruskal–Wallis test as appropriate. The significance level was assumed at 0.05. Regression analysis for a particular model of inheritance regarding the BDI score was performed.

## 3. Results

### 3.1. Sociodemographic Data

The study sample consisted of 1453 female respondents, the most numerous of whom were perimenopausal women (*n* = 708, 48.7%). The mean age of the women was 52.1 ± 7.9 years (min. 35, max. 81). More than half of the respondents (69.7%) lived in a city with a population of more than 100,000. The remaining ones lived in the city up to 100,000 inhabitants (15.5%), in the countryside (9.7%), and in the city up to 10,000 inhabitants (5.1%). The vast majority of the women were in a relationship (75.9%) and had third-level education (59.5%) or vocational education (40.5%). 78.3% of the participants were employed (Table 1).

We analyzed the relationship between menopausal stages according to WHO and selected sociodemographic variables (place of residence, marital status, education, and employment status). Based on the results, statistically significant differences in marital status, education, and employment status were observed between women at various menopausal stages. Third-level education was notably more common among premenopausal women (81%) than among their perimenopausal (60.7%) and postmenopausal (47%) counterparts. Moreover, the respondents’ employment status was significantly related to their age and menopausal status. The highest employment rate was observed among premenopausal women (96.4%), and then those in perimenopause (91.8%). In the case of postmenopausal women, half of the respondents worked, while the other half did not (Table 1).

### 3.2. Depressive Symptoms and the Genotypes of the 5-HTT and the MAO-A Gene Polymorphisms

We successfully genotyped for the 44-bp VNTR polymorphism in the 5-HTT gene promoter region and the 30-bp VNTR polymorphism in the MAO-A gene promoter region in all studied women. In the case of the 5-HTT gene, the number of women with particular genotypes were as follows: l/l = 557 (38.3%), l/s = 669 (46%), and s/s = 227 (15.6%). The genotype distribution for the MAO-A gene polymorphism was: 671 (46.2%) for the 3/4 genotype, 608 (41.8%) for the 4/4 genotype, and 174 (12%) for the 3/3 genotype. The prevalence of these polymorphisms met the Hardy–Weinberg equilibrium (chi2 = 1.23, *p* = 0.27; chi2 = 0.28, *p* = 0.59, respectively).

The following modes of genetic inheritance were analyzed to assess the influence of selected polymorphisms on the development and severity of depressive symptoms among the studied women:

- overdominant (SLC6A4: l/s vs. l/l + s/s; MAOA: 3/4 vs. 3/3 + 4/4);
- codominant (SLC6A4: l/l vs. l/s vs. s/s; MAOA: 3/3 vs. 3/4 vs. 4/4);
- recessive (SLC6A4: s/s vs. l/s + l/s; MAOA: 4/4 vs. 3/3+3/4); and
- dominant (SLC6A4: l/l vs. l/s +s/s; MAOA: 3/3 vs. 3/4+4/4).

We analyzed the relationship between the severity of depression according to the BDI and the frequency of the genotypes of the 44-bp VNTR polymorphism in the 5-HTT gene (SLC6A4) promoter region and the 30-bp VNTR polymorphism in the MAO-A gene (MAOA) promoter region.

Based on the results, we found that the severity of depressive symptoms according to the BDI (no depression, mild depression, moderate depression, and severe depression) was significantly related to the frequency of the genotypes of the 44-bp VNTR polymorphism in the 5-HTT gene promoter region (*p* < 0.05). In women without depressive symptoms and with moderate depressive symptoms, the l/l + l/s model was more common, while women with mild depressive symptoms had more often the l/s model (Table 2). When assessing the relationship between the frequency of the genotypes of the 44-bp VNTR polymorphism in the 5-HTT gene (SLC6A4) promoter region and the severity of depression according to the BDI, no statistically significant relationships were found (Table 2).

The analysis demonstrated statistically significant relationships between the frequency of the genotypes of the 30-bp VNTR polymorphism in the MAO-A gene promoter region and the severity of depression according to the BDI (*p* < 0.05). The recessive 4/4 genotype was more often found in women without depression than in those with depressive symptoms and depression. It was also observed that women without depressive symptoms were less often heterozygous (3/4). The analysis did not show any other statistically significant relationship between the frequency of the 30-bp VNTR polymorphism in the MAO-A gene promoter region and the severity of depression according to the Beck scale (Table 2).

### 3.3. Multiple Regression

To analyze a complex relationship between depression and genetic/environmental factors we conducted multivariable linear regression with regard to the BDI results (scores) as a dependent variable, as well as age, education, place of residence, and employment status as independent variables. Four modes of genetic inheritance were taken into account: codominant, recessive, dominant, and overdominant. The BDI scores were significantly associated with the codominant and recessive MAOA variants. The 4/4 genotype was associated with BDI scores lower by 1.27 (*p* = 0.032) and 0.96 (*p* = 0.008) for codominant and recessive models, respectively (Table 3). Linear regression demonstrated that the 5-HTT gene polymorphism was not related to the BDI scores.

### 3.4. Sociodemographic Data and Depressive Symptoms

The severity of depressive symptoms was assessed using the BDI. Based on the results, it was found that there were no cases of severe depression, and the majority of women had no depression at all (73%). Mild and moderate depression was observed in 24.6% and 2.6% of the respondents, respectively.

We analyzed the relationship between depressive symptoms and the stage of menopause. The results revealed a statistically significant relationship. Perimenopausal women were much more likely to be non-depressive than the rest of the respondents, while premenopausal women were less likely to be moderately depressed. The study also showed a statistically significant relationship between the scores obtained on the BDI and the stage of menopause. Premenopausal women scored significantly lower on the BDI than the rest of the surveyed women.

We assessed the influence of sociodemographic variables (place of residence, marital status, education, and employment status) on the level of depression among the studied women. It was shown that employed women much more often did not show any signs of depression than unemployed ones. No statistically significant relationships were observed between the severity of depressive symptoms and other sociodemographic variables (Table 4).

We also analyzed the influence of sociodemographic variables (place of residence, marital status, education, and employment status) on the BDI scores. The BDI scores were statistically significantly related to employment status and the stage of menopause according to WHO―employed respondents scored lower on the BDI than unemployed ones, and premenopausal women scored lower than their peri- and postmenopausal counterparts. In the case of the remaining sociodemographic variables, no statistically significant relationships with the BDI scores were observed. Severe depression was not included in the table, because none of the respondents obtained more than 26 points on the BDI (Table 4).

## 4. Discussion

Although menopause is the normal, natural transition in life, it substantially contributes to problems related to physical functioning, as well as mood disorders, including depressive symptoms [57]. The severity of depression is significantly influenced by the stage of menopause [14,15,16,17,18]―many scientists emphasize that the most difficult stage in this respect is perimenopause. Our research has confirmed that premenopausal women usually do not experience symptoms of depression, and that the severity of depressive symptoms significantly increases in the postmenopausal period. In our study, depressive symptoms were found in 27% of women. Similar results were obtained by Grochans et al. [58], who observed moderate and severe depressive symptoms according to the BDI in 25.5% of the Polish women aged 45–60 years.

### 4.1. Depressive Symptoms and the Genotypes of the 5-HTT and MAO-A Gene Polymorphisms

A 2002 study by Caspi et al. [59], whose results were later confirmed by several other researchers, showed that a genotype predisposing to high *MAO-A* activity in women who have experienced abuse promotes the development of aggressive and antisocial behavior. Interestingly, an inverse relationship was demonstrated in men: carrying the genotype associated with low *MAO-A* activity resulted in stronger pathological tendencies [60,61,62,63,64,65,66,67]. The study conducted by Nilsson et al. in 2018 [68] suggests that the MAO-A gene polymorphism may affect the degree of emotion regulation. The combination of a highly active genotype with negative environmental influences may result in a higher degree of emotional reactivity in women, and so can be treated as a predictor of the occurrence of pathological behaviors [69].

It has been shown that there is a negative correlation between emotional stability and the severity of depressive symptoms. This relationship is particularly evident in women, who are generally characterized by a higher degree of neuroticism than men [70,71]. Research by Rodríguez-Ramos et al. [72] revealed that the presence of the high activity MAOA genotype entails a higher level of neuroticism in healthy women than the genotype with lower reactivity. Yu et al. demonstrated that harm avoidance is typical of women with a genotype predisposing to higher *MAO-A* activity, which is associated with a higher susceptibility to depression and anxiety disorders [73]. In the study of menopausal women conducted by Esmaeilzadeh et al. no relationship between the *MAO-A* gene polymorphism and the severity of depression and depressive symptoms was found [74]. Our study showed that women with a recessive or overdominant model of MAOA inheritance may be at increased risk of developing depressive disorders. Multivariable regression with regard to the BDI scores, age, education, place of residence, employment status, and all models of inheritance for the polymorphisms analyzed demonstrated that the 4/4 MAO genotype was associated with a lower risk of depressive disorders. This stands in contrast to the results reported by other authors.

Many researchers indicate an important role of serotonin in regulating the functioning of the central nervous system. It has been shown that the level of serotonin may play an important part in the development of depression [75,76] and affect the expression of personality traits [77]. The *5-HTT* gene polymorphism may increase the risk of developing depression, especially among people who have been exposed to stressful life events [56,78,79,80]. Some studies also highlighted the relationship between the *5-HTT* gene polymorphism and personality traits predisposing to affective disorders, such as neuroticism and impulsiveness―people with the “s” allele are more likely to develop such disorders. This relationship has been confirmed in alcoholics [45,46,81], people attempting suicide [44,82] and girls who, being exposed to adverse effects of the home environment, exhibited a higher degree of pathological reactions compared to carriers of the “l” allele [83]. In the light of these reports, it seems reasonable to assume that the *5-HTT* gene polymorphism may have a direct impact on the occurrence of mood disorders, the level of anxiety, and stress resistance [78]. The studies conducted among perimenopausal women, without taking into account the exposure to adverse environmental conditions, did not confirm the existence of a relationship between the *5-HTT* gene polymorphism and depression [83] or the level of anxiety [84]. This is in line with the results of our research―no clear relationship has been found between different models of genetic inheritance and the level of depressive symptoms measured by the BDI.

### 4.2. Sociodemographic Data and Depressive Symptoms

Many studies indicate a significant relationship between the stage of menopause and the risk of depressive symptoms in women [32,85,86]. Freeman et at. [34] demonstrated that perimenopausal women were at over four-time higher risk of clinically significant depressive symptoms than premenopausal ones. Similar results were obtained in the Harvard Study of Moods and Cycles [87]. Mulhall et al. [17] also confirmed the increased probability of depressive symptoms in perimenopause compared to premenopause but emphasized that this phenomenon is independent of socio-demographic factors, lifestyle, and women’s health. Different conclusions were drawn by Woods et al. [88], who believe that depressive symptoms do not depend on the stage of menopause, and that the development of depressive symptoms in women is influenced by many variables.

Some researchers claim that the severity of depressive symptoms in the menopausal period is determined by sociodemographic factors [89,90,91], life stress, and health problems [88]. Many studies prove the positive impact of higher education [32,92,93,94], which is associated with better knowledge, cognitive development, and greater social support. All these elements provide a means of responding effectively to stressful life events, and thus reduce the frequency of depressive symptoms [95,96,97,98]. Scientists note that having a partner [96] and a job [93,97] has a positive effect on women’s mental health. Different conclusions were drawn by Campbell et al. [98], who found that marital status, employment, and education did not affect the level of depression in menopausal women. In our study, both the stage of menopause and employment status had a significant effect on the development of depressive symptoms. Working women experienced severe depressive symptoms much less often than their non-working counterparts.

## 5. Conclusions

Our research on a large group of middle-aged women showed that depressive symptoms affect more than one in four of them. The factors that predispose middle-aged women to depressive symptoms are unemployment and the postmenopausal stage.

It has been shown that respondents with the l/s genotype of the 44-bp polymorphism in the *5-HTT* gene (SLC6A4) promoter region were more likely to suffer from mild depressive symptoms according to the BDI, while in women without, or with, moderate depressive symptoms, the l/l + l/s model was more often found. Moreover, the analysis of the 30-bp VNTR polymorphism in the MAOA promoter region demonstrated that in women with depressive symptoms according to the BDI, the 3/3 dominant genotype and the 3/3 + 3/4 recessive genotype were more common compared to women without depressive symptoms.

Multivariate regression analysis did not reveal any relationship between the models of inheritance for *5-HTT* gene polymorphisms and depressive symptoms in middle-aged women.

## 6. Limitations of the Study

The contribution of genetic and sociodemographic factors to depressive symptoms in women was analyzed among 1453 healthy Polish women at different stages of menopause. In our research, descriptive questionnaires were used to assess depressive symptoms, in which the respondents assessed their symptoms themselves. It is necessary to take into account the possibility of limited objectivity of the results due to their declarative character.

Amenorrhea for at least 12 months was diagnosed on the basis of gynecological history but was not confirmed by the measurement of the FSH level. Both of these variables may have influenced the results of our research.

## Figures and Tables

**Table 1:** The structure of the study sample with regard to socio-demographic data, age, and the stage of menopause according to WHO.

Sociodemographic Data		Age of the Respondents [Years]			Stage of Menopause According to WHO			
		Me	IQR	*p*	Premenopause (*n* = 247)	Perimenopause (*n* = 708)	Postmenopause (*n* = 498)	*p*
Place of residence	rural areas (*n* = 141)	51	11	0.398	22 (9%)	77 (11%)	42 (8%)	0.5
	city up to 10,000 inhabitants (*n* = 74)	52.5	9		11 (4%)	39 (5%)	24 (5%)	
	city up to 100,000 inhabitants (*n* = 225)	53	9		31 (13%)	112 (16%)	82 (17%)	
	city of more than 100,000 inhabitants (*n* = 1013)	53	11		183 (74%)	480 (68%)	350 (70%)	
Marital status	formal relationship (*n* = 1046)	52	10	0.097	178 (72%)	527 (74%)	341 (68%)	<0.0001
	informal relationship (*n* = 57)	52	6		2 (1%)	42 (6%)	13 (3%)	
	single (*n* = 350)	54	13		67 (27%)	139 (20%)	144 (29%)	
Education	primary (*n* = 1)	54	0	<0.000001	0 (0%)	1 (0.2%)	0 (0%)	<0.0001
	vocational (*n* = 588)	55	9		47 (19%)	277 (39.1%)	264 (53%)	
	third-level (*n* = 864)	51	11		200 (81%)	430 (60.7%)	234 (47%)	
Employment status	yes (*n* = 1137)	51	8	<0.0001	238 (96%)	650 (92%)	249 (50%)	<0.0001
	no (*n* = 316)	60	10		9 (4%)	58 (8%)	249 (50%)	

Me―median, IQR―interquartile range, *p*―statistical significance.

**Table 2:** The genotype distribution for the 5-HTT and MAO-A gene polymorphisms and the severity of depressive symptoms according to the Beck Depression Inventory (BDI).

Genes		Severity of Depression According to the BDI				Severity of Depression According to the BDI			Severity of Depression According to the BDI (Score)		
		No Depression (*n* = 1061)	Mild Depression (*n* = 358)	Moderate Depression (*n* = 34)	*p*	No Depression (*n* = 1061)	Depressive Symptoms and Depression (*n* = 392)	*p*	Me	IQR	*p*
Recessive MAO-A	3/3+3/4	597 (70.7%)	226 (26.7%)	22 (2.6%)	0.0553	597 (70.7%)	258 (29.3%)	0.0164	8	9	0.00564
	4/4	464 (76.3%)	132 (21.7%)	12 (2%)		464 (76.3%)	144 (23.7%)		7	8	
Dominant MAO-A	4/4+3+4	937 (73.3%)	315 (24.6%)	27 (2.1%)	0.2899	937 (73.3%)	342 (26.7%)	0.578	8	8	0.361545
	3/3	124 (71.3%)	43 (24.7%)	7 (4%)		124(71.3%)	50 (28.7)		7	10	
Overdominant MAO-A	3/3+4/4	588 (75.2%)	175 (22.4%)	19 (2.4%)	0.097	588(75.2%)	194 (24.8%)	0.0443	7	8	0.031996
	3/4	473 (70.5%)	183 (27.3%)	15 (2.2%)		473 (70.5%)	198 (29.5%)		8	9	
Codominant MAO-A	3/3	124 (71.3%)	43 (24.7%)	7 (4%)	0.0895	124 (71.3%)	50(28.7%)	0.055	7	7	0.49688
	3/4	473 (70.5%)	183 (27.3%)	15 (2.2%)		473(70.5%)	198 (29.5%)		8	9	
	4/4	464 (76.3%)	132 (21.7%)	12 (2%)		464 (76.3%)	144(23.7%)		7	9	
Recessive 5-HTT	l/l+l/s	898 (73.2%)	54 (23.8%)	24(2.0%)	0.0806	898 (73.2%)	328 (26.8%)	0.6535	7	8	0.797784
	s/s	163 (71.8%)	304 (24.8%)	10 (4.4%)		163(71.8%)	64 (28.2%)		7	9	
Dominant 5-HTT	l/s+s/s	640 (71.4%)	235 (26.2%)	21 (2.3%)	0.2	640 (71.4%)	256 (28.6%)	0.0829	8	8	0.317583
	l/l	421 (75.6%)	123 (22.1%)	12 (2.3%)		421 (75.6%)	136(24.4%)		7	7	
Overdominant 5-HTT	l/l+l/s	584 (74.5%)	177 (22.6%)	23 (2.9%)	0.0496	584 (74.5%)	200 (25.5%)	0.1723	7	8	0.245501
	l/s	477 (71.3%)	181 (27.1%)	11 (1.6%)		477(71.3%)	192 (28.7%)		8	9	
Codominant 5-HTT	l/l	421 (75.6%)	123 (22.1%)	13 (2.3%)	0.0504	421 (75.6%)	136(24.4%)	0.2196	7	7	0.496875
	l/s	477 (71.3%)	181 (27.1%)	11 (1.6%)		477 (71.3%)	192 (28.7%)		8	9	
	s/s	163 (71.8%)	54 (23.8%)	10 (4.4%)		163(71.8%)	64 (28.2%)		7	9	

Me―median, IQR―interquartile range, *p*―statistical significance.

**Table 3:** Regression analysis between genetic models and depressive symptoms according to the BDI.

Questionnaire	Gene		Genetic Models Used in Regression Analysis			
			Codominant	Recessive	Dominant	Overdominant
**BDI**	***MAO-A***	coefficient	−1.27	−0.96	−0.81	0.60
		t	−2.14	−2.64	−1.45	1.66
		*p*	0.032	0.008	0.148	0.097
	***5-HTT***	coefficient	ns	ns	ns	ns
		t	ns	ns	ns	ns
		*p*	ns	ns	ns	ns

ns- no significant.

**Table 4:** Association between the severity of depressive symptoms according to the BDI and sociodemographic data.

Genes		Severity of Depression According to the BDI				Occurrence of Depression According to the BDI			Severity of Depression According to the BDI (Score)		
		No Depression (*n* = 1061)	Mild Depression (*n* = 358)	Moderate Depression (*n* = 34)	*p*	No Depression (*n* = 1061)	Depressive Symptoms and Depression (*n* = 392)	*p*	Me	IQR	*p*
Premenopause (*n* = 247)		199 (80.6%)	44 (17.8%)	4 (1.6%)	0.0019	199 (80.6%)	48 (19.4%)	0.0004	5	8	<0.000001
Perimenopause (*n* = 708)		526 (74.3%)	163 (23%)	19 (2.7%)		526 (74.3%)	182 (25.7%)		8	8	
Postmenopause (*n* = 498)		336 (67.5%)	151 (30.3%)	11 (2.2%)		336 (67.5%)	162 (32.5%)		8	8	
Place of residence	rural areas (*n* = 141)	113 (80.1%)	25 (17.7%)	3 (2.1%)	0.3	113 (80.1%)	28 (19.8%)	0.1871	7	7	0.4
	city up to 10,000 inhabitants(*n*=74)	55 (74.3%)	19 (25.7%)	0 (0%)		55 (74.3%)	19 (25.7%)		7	8	
	city up to 100,000 inhabitants (*n* = 225)	167 (74.2%)	54 (24%)	4 (1.4%)		167 (74.2%)	58 (25.4%)		7	8	
	city of more than 100,000 inhabitants (*n* = 1013)	726 (71.7%)	260 (25.7%)	27 (2.7%)		726 (71.7%)	287 (28.3%)		8	9	
Marital status	formal relationship (*n* = 1046)	781(74.7%)	238 (22.8%)	27 (2.6%)	0.06	781 (74.7%)	265 (25.4%)	0.0516	7	8	0.1
	informal relationship (*n* = 57)	42(73.7%)	15 (26.3%)	0 (0%)		42 (73.7%)	15 (26.3%)		7	7.25	
	single (*n* = 350)	238 (68%)	105 (30%)	7 (2%)		238 (68%)	112 (32%)		8	9	
Employment status	Yes (*n* = 1137)	860 (75.6%)	252 (22.2%)	25 (2.2%)	0.0001	860 (75.6%)	277 (24.4%)	<0.0001	7	8	0.0004
	No (*n* = 316)	201 (63.6%)	106 (33.5%)	9 (2.8%)		201 (63.6%)	115 (36.3%)		8	10	
Education	primary (*n* = 1)	1 (100%)	0 (0%)	0 (0%)	0.88	1 (100%)	0 (0%)	0.8131	2	0	0.3
	vocational (*n* = 588)	431 (73.3%)	141 (24%)	16 (2.7%)		431 (73.3%)	157 (26.7%)		7	8	
	third-level (*n* = 864)	629 (72.8%)	217 (25.1%)	18 (2.1%)		629 (72.8%)	235 (27.2%)		7	9	

Me―median, IQR―interquartile range, *p*―statistical significance.

## Data Availability

Data available on request due to restrictions ethical.
